# *Bifidobacterium breve* with α-Linolenic Acid and Linoleic Acid Alters Fatty Acid Metabolism in the Maternal Separation Model of Irritable Bowel Syndrome

**DOI:** 10.1371/journal.pone.0048159

**Published:** 2012-11-20

**Authors:** Eoin Barrett, Patrick Fitzgerald, Timothy G. Dinan, John F. Cryan, R. Paul Ross, Eamonn M. Quigley, Fergus Shanahan, Barry Kiely, Gerald F. Fitzgerald, Paul W. O'Toole, Catherine Stanton

**Affiliations:** 1 Alimentary Pharmabiotic Centre, Biosciences Institute, University College Cork, Cork, Ireland; 2 Teagasc, Food Research Centre, Moorepark, Fermoy, Co. Cork, Ireland; 3 Department of Microbiology, University College Cork, Cork, Ireland; Laurentian University, Canada

## Abstract

The aim of this study was to compare the impact of dietary supplementation with a *Bifidobacterium breve* strain together with linoleic acid & α-linolenic acid, for 7 weeks, on colonic sensitivity and fatty acid metabolism in rats. Maternally separated and non-maternally separated Sprague Dawley rats (n = 15) were orally gavaged with either *B. breve* DPC6330 (10^9^ microorganisms/day) alone or in combination with 0.5% (w/w) linoleic acid & 0.5% (w/w) α-linolenic acid, daily for 7 weeks and compared with trehalose and bovine serum albumin. Tissue fatty acid composition was assessed by gas-liquid chromatography and visceral hypersensitivity was assessed by colorectal distension. Significant differences in the fatty acid profiles of the non-separated controls and maternally separated controls were observed for α-linolenic acid and arachidonic acid in the liver, oleic acid and eicosenoic acid (*c*11) in adipose tissue, and for palmitoleic acid and docosahexaenoic acid in serum (p<0.05). Administration of *B. breve* DPC6330 to MS rats significantly increased palmitoleic acid, arachidonic acid and docosahexaenoic acid in the liver, eicosenoic acid (*c*11) in adipose tissue and palmitoleic acid in the prefrontal cortex (p<0.05), whereas feeding *B. breve* DPC6330 to non separated rats significantly increased eicosapentaenoic acid and docosapentaenoic acid in serum (p<0.05) compared with the NS un-supplemented controls. Administration of *B. breve* DPC6330 in combination with linoleic acid and α-linolenic acid to maternally separated rats significantly increased docosapentaenoic acid in the serum (p<0.01) and α-linolenic acid in adipose tissue (p<0.001), whereas feeding *B. breve* DPC6330 with fatty acid supplementation to non-separated rats significantly increased liver and serum docosapentaenoic acid (p<0.05), and α-linolenic acid in adipose tissue (p<0.001). *B. breve* DPC6330 influenced host fatty acid metabolism. Administration of *B. breve* DPC6330 to maternally separated rats significantly modified the palmitoleic acid, arachidonic acid and docosahexaenoic acid contents in tissues. The effect was not observed in non-separated animals.

## Introduction

The microbiota of the gastrointestinal (GI) tract contains trillions of microorganisms performing vital functions for the host [1], [2]. The GI microbiota has a significant influence on human health and has been implicated in a number of disease states, including obesity and inflammatory bowel diseases [3], [4]. Of particular interest among the gut microbiota are the commensal group, *Bifidobacterium*, which constitute an estimated 3% of the intestinal microbiota of adults [5], [6] and can dominate and outnumber all other bacterial groups and species in newborns [7], [8], [9]. They are considered, along with lactobacilli, to be among the most important health promoting bacteria in humans, as well as being among the most studied probiotics [10], [11]. Proposed health benefits include reductions of diarrhoea, rotavirus infection, and atopic dermatitis, amelioration of lactose intolerance, and modulation of the immune system [12], [13], [14]. One further proposed health benefit is alleviation of the symptoms of irritable bowel syndrome (IBS), thus extensive research has been conducted on the use of bifidobacteria and lactic acid bacteria in the treatment of this disorder [15], [16], [17].

IBS is a common functional disorder of the human GI tract, characterised by abdominal pain and discomfort, and is generally viewed as a disorder of the brain-gut axis [18], [19]. It is one of the most common reasons for patient visits to gastroenterologists and has an estimated worldwide prevalence of 10–15% [19]. An inability to cope with stress has been implicated in the development and aggravation of IBS, in addition to immune activation, alterations in the gut microbiota and visceral hypersensitivity [20], [21], [22]. One such stress is early life maternal separation, which has been implicated in alterations in the development of the central nervous system [23], [24], [25]. Early life stress in humans predisposes individuals to stress related disorders such as IBS later in life [26], [27]. This can be readily modelled in animals using the maternal separation model whereby brief separation of rat pups from the mother triggering long-term changes in colonic sensitivity to rectal distension, which manifests itself as an increase in visceral pain, thereby mimicking the clinical features of IBS [25], [28]. It has also been reported that the ω-6: ω-3 fatty acid ratio in maternally separated rodents was significantly increased when compared with non-separated rodents [29]. For example, arachidonic acid (C20:4n-6), a fatty acid found in elevated concentrations in the plasma of IBS patients, was significantly elevated in plasma of separated animals compared with control rodents [29]. In addition to altered fatty acid ratio, it has also been reported that feeding *B. infantis* reverses behavioural defects in maternally separated rats [30].

Essential fatty acids are important components of all neuronal membranes and perform vital roles in regulating the activity of both ionotropic and metabotropic receptors [31]. α-Linolenic acid (ALA, C18:3n-3), a precursor of ω-3 series fatty acids, and *cis*-linoleic acid (LA, C18:2n-6), a precursor of ω-6 series fatty acids are essential fatty acids in humans [32]. ALA is converted to eicosapentaenoic acid (EPA, C20:5n-3) which in turn is converted to the 3-series of prostaglandins and docosahexaenoic acid (DHA, C22:6n-3). LA is converted to γ-linolenic acid (GLA, C18:3n-6) which can be metabolized to dihomo-GLA (C20:3n-6) and arachidonic acid, precursors of the 1- and 2- series of prostaglandins. Indeed, it has been reported that the aforementioned fatty acids can influence pro-inflammatory cytokine production, with EPA and DHA inhibiting the production of tumour necrosis factor-α (TNF-α), interleukin (IL)-6 and IL-2, while arachidonic acid increases the production of pro-inflammatory eicosanoids and consequently pro-inflammatory cytokines [33].

The ω-6: ω-3 fatty acid ratio is an important factor in health, with an ideal diet incorporating a dietary ratio of 4∶1 ω-6 and ω-3, respectively [34]. Most Western diets have a 10∶1 ratio in favour of ω-6 polyunsaturated fatty acids, predominantly due to increased LA consumption [35], and high levels of coronary artery disease, some cancers and possibly depression have been attributed to this imbalance [36], [37]. Furthermore, patients suffering from IBS have been reported to have elevated plasma arachidonic acid, in addition to elevated plasma IL-6 [38], [39], [40], and more recently lower levels of arachidonic acid have been detected in the serum of IBS patients [41].

A possible way of altering the ω-6: ω-3 dietary imbalance is by modulation with conjugated linoleic acid (CLA), a natural component of ruminant milk and tissue fat. It comprises of a mixture of positional and geometric conjugated isomers of LA and has a number of proposed health benefits, most notably anti-carcinogenic, immuno-modulatory, anti-obesity and anti-atherosclerotic activities [42], [43], [44], [45]. It has been reported that CLA can induce essential fatty acid redistribution in mice [46]. Alterations in DHA and arachidonic acid levels were observed in some organs. For example, the DHA content of heart tissue was reduced by 25%, while in the spleen, DHA content increased and arachidonic acid content was reduced [46]. Furthermore, feeding CLA producing bifidobacteria can influence the fatty acid composition of murine tissues [47], [48]. It has been reported that the CLA-producing bacterium, *B. breve* NCIMB702258, converts LA to CLA in the murine gut, resulting in significantly elevated *c*9, *t*11 CLA in the liver [47]. Significantly higher concentrations of EPA, DHA, dihomo-γ-linolenic acid (20:3n-6) and stearic acid (18∶0) were also reported in the colon, as well as significantly higher concentrations of stearidonic acid (18:4n-3) in liver and DHA in brain [47], [49].

The aim of the current study was to examine the impact of dietary intervention with *B. breve* DPC6330 together with 0.5% (w/w) LA & 0.5% (w/w) ALA on visceral sensitivity and fatty acid metabolism in maternally separated adult rats.

## Materials and Methods

### Animals

Two groups, maternally separated (MS) (n = 45) and non-separated (NS) (n = 45), of Sprague Dawley (Harlan Ltd. Briester, UK) rat pups were used in this study. The pups were housed with their mothers in plastic cages in a temperature controlled room on 12 h light/dark cycle. Each of the two groups were subsequently divided into 3 groups (n = 15) (6×15) and assigned to one of the following dietary treatments: One group received 0.5% (w/w) LA plus 0.5% (w/w) ALA (triglyceride bound form; Larodan Fine Chemicals, Malmo, Sweden) of their diet together with 1×10^9^ live *B. breve* DPC6330 microorganisms daily. A second group received 1×10^9^ live *B. breve* DPC6330 microorganisms, while the third group received a placebo (15% (w/v) trehalose added as a cryoprotectant plus 2% (w/v) bovine serum albumin added as an emulsifier). The treatments were administered by oral gavage for 7 weeks starting at postnatal day (PND) 28. The diet was sterilized before feeding and contained the following nutrient composition: crude protein (18.6%), fat (6.2%), crude fiber (3.5%), neutral detergent fiber (14.7%), and ash (5.3%). The fatty acids present in the diet included palmitic acid (C16:0, 0.7%), stearic acid (C18:0, 0.2%), oleic acid (C18:1n-9, 1.2%), LA (C18:2n-6, 3.1%), and ALA (C18:3n-3, 0.3%). Body weight was assessed weekly. Following 7 weeks on experimental diets, the animals were sacrificed by decapitation. Liver, prefrontal cortex, and mesenteric adipose tissue were removed, blotted dry on filter paper, weighed and flash-frozen immediately in liquid nitrogen. Blood serum was collected by allowing blood samples to clot for 2 h at 4°C before centrifuging at 3000× g for 20 min and collecting the serum. All samples were stored at −80°C until processed.

### Preparation and administration of *B. breve* DPC6330

*B. breve* DPC6330, which has previously been shown to convert ≤80% LA to *c*9, *t*11 CLA when grown in 0.5 mg/ml LA *in vitro* was stocked at −80°C in 40% (v/v) glycerol in the Teagasc, Moorepark Food Research Centre culture collection. The genome sequence of the strain has been elucidated [50]. The strain was cultured in MRS broth (Difco, Detroit, Mich.) supplemented with 0.05% (w/v) L-cysteine-hydrochloride (mMRS) (98% pure Sigma Chemical Co., St. Louis, Mo.) under anaerobic (anaerobic jars with Anaerocult®A gas packs; Merck, Darmstadt, Germany) conditions at 37°C. When a solid medium was required, 1.5% (w/v) agar (Oxoid, Hampshire, UK) was added to the mMRS medium. For use in the rat trials, *B. breve* DPC6330 was initially grown in mMRS for 72 h, washed twice in phosphate buffered saline (PBS) and resuspended at ∼1×10^10^ cells/ml in 15% (w/v) trehalose (Sigma). Two hundred ml volumes were freeze-dried using a 24 h program (freeze temperature −40°C, condenser set point −60, vacuum set point 1.33×10^−3^ mBarr). The number of *B. breve* DPC6330 colony forming units (CFU) per gram of freeze dried powder was determined by serially diluting 1 gram of powder in maximum recovery diluent (MRD) (Oxoid) and plating on mMRS agar at 37°C under anaerobic conditions.

### Separation Procedure

Rat pups were separated from their mother for 3 hours every day from post natal day (PND) 2 to 12 as previously described [25]. From PND 13 until 22, pups were maintained with their mothers. All animals were weaned on PND 22.

### Colorectal Distension

Each animal was anaesthetized with isoflourane and a latex balloon, 6 cm in length was inserted into the colon, 1 cm from the anus. The animals recovered for 10 min prior to colorectal distension (CRD). The balloon was then connected to a barostat and the animal placed in an observation chamber. The balloon was distended from 0–80 mm Hg over an 8 min period. During this time, the threshold i.e. the pressure at which the animals displayed their first pain behaviour, as well as the number of pain behaviours were noted. The frequency of contractions of the abdominal wall is considered as a reliable marker of visceral sensitivity [25]. Animals were tested in a random fashion and the experimenter was blinded to the individual groups.

### Lipid extraction and fatty acid analysis

Lipids were extracted using chloroform:methanol (2∶1 v/v; Fisher Scientific, Dublin, Ireland) according to a previously described method [51]. Fatty acid methyl esters (FAME) were prepared by adding 10 ml 0.5 N NaOH (Sigma) in methanol to dry fat for 10 min at 90°C followed by 10 ml 14% (w/v) BF_3_ in methanol (Sigma) for 10 min at 90°C (21). FAME were extracted with hexane (Fisher Scientific), dried with 0.5 g of anhydrous sodium sulphate (Sigma) for 1 hr and stored at −20°C prior to gas-liquid chromatography (GLC) analysis. FAME were separated by GLC (Varian 3800, Varian, Walnut Creek, CA, USA) fitted with a flame ionization detector, using a Chrompack CP Sil 88 column (Chrompack, Middleton, The Netherlands, 100 m×0.25 mm i.d., 0.20 µm film thickness) and Helium as carrier gas. The column oven was programmed to be held initially at 80°C for 8 min then increased 8.5°C/min to a final column temperature of 200°C. The injection volume used was 0.6 µl, with automatic sample injection on a SPI 1093 splitless on-column temperature programmable injector. Peaks were integrated using the Varian Star Chromatography Workstation version 6.0 software and peaks were identified by comparison of retention times with pure FAME standards (Nu-Chek Prep, Elysian, MN, USA). The percentage of individual fatty acids was calculated according to the peak areas relative to the total area (total fatty acids were set at 100%). All fatty acid results are shown as mean ± standard error of the mean (SEM) g/100 g FAME.

All laboratory animal experimentations were performed according to the guidelines for the care and use of laboratory animals approved by the Department of Health and Children of the Irish government. This study was approved by the ethics committee of University College Cork.

### Statistical analysis

Results in the text, tables and figures are presented as Mean per group ± SEM. To assess if differences between treatment groups were significant, data were analysed using one-way analysis of variance (ANOVA) followed by post hoc Tukey's multiple comparison tests using GraphPad Prism version 5.0 for Windows (GraphPad Software, San Diego, CA, USA). Results were considered significant as follows: * p<0.05, **p<0.01, ***p<0.001. Data were analysed for outliers using Grubb's test (GraphPad Software).

## Results

There was no significant difference in the rate of weight gain between the groups (data not shown).

### The effect of *B. breve* DPC6330±0.5% (w/w) linoleic acid and 0.5% (w/w) α-linolenic acid on visceral hypersensitivity

**Figure 1:**
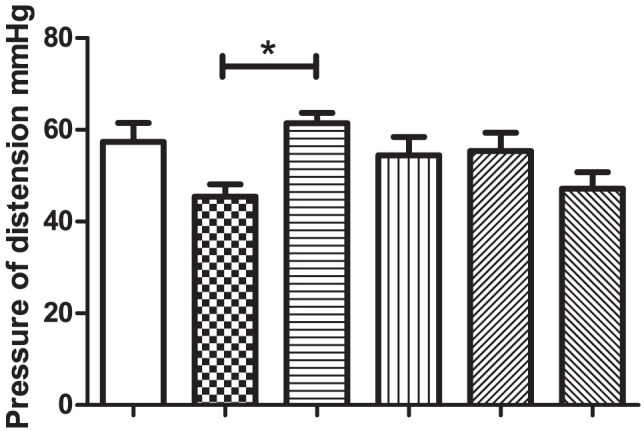
The average threshold value (pressure at which the animal shows the first pain behaviour) of each group. (* = p<0.05). White columns- Non separated rats fed un-supplemented diet, Chequered columns- Maternally separated fed un-supplemented diet, Horizontal lines- Non separated rats fed *Bifidobacterium breve* DPC 6330, Vertical lines- Maternally separated rats fed *Bifidobacterium breve* DPC 6330, Upward diagonal lines- Non separated rats fed *Bifidobacterium breve* DPC 6330 plus 0.5% linoleic acid and 0.5% α-linolenic acid, Downward diagonal lines- Maternally separated rats fed *Bifidobacterium breve* DPC6330 plus 0.5% (w/w) linoleic acid and 0.5% (w/w) α-linolenic acid.

While the MS group fed an un-supplemented diet exhibited the lowest pain threshold of all six groups included in this study, the NS rats fed *B. breve* DPC6330 were the only group which displayed an increased pain threshold compared to the MS control group (p<0.05, Figure 1).

### The effect of *B. breve* DPC6330±0.5% (w/w) linoleic acid and 0.5% (w/w) α-linolenic acid on the liver fatty acid composition

Following 7 weeks of dietary treatment, the MS control group had the lowest DHA content in the liver (Table 1). Administration of *B. breve* DPC6330 to MS rats led to significantly increased DHA content in the liver, compared with the un-supplemented control group (p<0.01, Table 1). The NS rats receiving *B. breve* in combination with LA and ALA had a higher concentration of DPA in the liver after 7 weeks of feeding compared to the MS and NS controls (p<0.05 Table 1), with the MS control group having the lowest amount of DPA in the liver of all the groups (p<0.05). The mean arachidonic acid content of the liver was significantly higher in the NS control rats compared to the MS control rats (p<0.05, Table 1). Indeed, the MS control rats had lowest concentration of arachidonic acid and highest concentration of palmitoleic acid in the liver of all the groups. Oral administration of *B. breve* DC6330 also led to a significant increase in the arachidonic acid content of MS rats and a significant decrease in palmitoleic acid (p<0.05, Table 1). Higher amounts of ALA were found in the liver of the MS control group compared to NS control group (p<0.05 Table 1).

**Table 1:** Fatty acid profile in liver of Non-Maternally Separated rats fed un-supplemented diet (A), Maternally Separated rats fed un-supplemented diet (B), Non-Maternally Separated rats fed *B. breve* DPC6330 (C), Maternally Separated rats fed *B. breve* DPC6330 (D), Non-Maternally Separated rats fed *B. breve* DPC6330 plus 0.5% (w/w) linoleic acid and 0.5% (w/w) α-linolenic acid (E), Maternally Separated rats fed *B. breve* DPC6330 plus 0.5% (w/w) linoleic acid and 0.5% (w/w) α-linolenic acid (F) for 7 weeks.

g/100 g FAME	Liver					
	A	B	C	D	E	F
**C16:0**	31.98±0.48	32.55±0.33	31.84±0.74	32.52±0.36	32.28±0.61	31.92±0.45
**C16:1c9**	2.08±0.25	^d*^ 2.83±0.25	2.28±0.23	^b*^ 1.91±0.19	2.30±0.17	2.17±0.17
**C18:0**	13.63±0.36	14.18±0.33	13.83±0.52	13.17±0.30	14.10±0.53	14.36±0.48
**C18:1c9**	6.26±0.16	6.93±0.19	6.44±0.18	6.28±0.22	6.46±0.16	6.33±0.14
**C18:1** ***t*** **9**	2.15±0.06	2.55±0.10	2.25±0.13	2.13±0.10	2.31±0.42	2.19±0.10
**C18:2** ***n*** **-6**	17.38±0.22	17.06±0.25	16.70±0.43	16.53±0.47	16.32±0.37	16.44±0.28
**C18:3** ***n*** **-3**	^b*^ 0.309±0.01	^a*^ 0.372±0.02	0.330±0.01	0.329±0.01	0.343±0.01	0.328±0.01
**C20:4** ***n*** **-6**	^b*^ 14.06±0.24	^a*,d*^ 12.76±0.32	13.13±0.25	^b*^ 14.02±0.27	13.43±0.21	13.42±0.35
**C22:5** ***n*** **-3**	^e*^ 0.776±0.04	^e*^ 0.762±0.06	0.904±0.06	0.878±0.04	^a*,b*^ 1.025±0.05	0.931±0.04
**C22:6** ***n-3***	3.26±0.14	^d**,e*^ 2.62±0.12	2.89±0.08	^b**^ 3.41±0.21	^b*^ 3.31±0.73	3.09±0.10

Results are expressed as percentage of total identified fatty acids. Data are Means ± SEM g/100 g FAME. ^A, B, C, D, E, F^ Different superscript letters within a column indicate significant difference (* = p<0.05, ** = p<0.01, *** = p<0.001). FAME = fatty acid methyl esters. C16:0 palmitic acid; C16:1c9 palmitoleic acid; C18:0 stearic acid; C18:1c9 oleic acid; C18:2n-6 linoleic acid; C18:3n-3 linolenic acid; C20:4n-6 arachidonic acid; C22:5n-3 docosapentaenoic acid; C22:6n-3 docosahexaenoic acid.

### The effect of *B. breve* DPC6330±0.5% (w/w) linoleic acid and 0.5% (w/w) α-linolenic acid on the adipose tissue fatty acid composition

The palmitoleic acid content of the adipose tissue of MS control rats was the lowest of all six groups after 7 weeks of dietary treatment. Furthermore, only the NS rats receiving *B. breve* DPC6330 had a significantly higher palmitoleic acid content in the adipose tissue compared with the MS group (p<0.05, Table 2). The MS control group had significantly lower oleic acid than the NS control group (p<0.05, Table 2). The MS control group also exhibited decreased oleic acid content of adipose tissue when compared with the NS rats receiving *B. breve* DPC6330, however, feeding *B. breve* DPC6330 did not increase oleic acid in NS rats (Table 2). The MS and NS groups receiving *B. breve* with LA and ALA supplementation (groups E and F) exhibited higher concentrations of ALA (p<0.001) in adipose tissue compared to un-supplemented or *B. breve* DPC6330 supplemented rats (Table 2). The MS control group had significantly higher eicosenoic acid (C20:1*c*11) than the NS control group (p<0.05, Table 2). Administration of *B. breve* DPC6330 to MS rats decreased eicosenoic acid (p<0.05, Table 2).

**Table 2:** Fatty acid profile in adipose tissue of Non-Maternally Separated rats fed un-supplemented diet (A), Maternally Separated rats fed un-supplemented diet (B), Non-Maternally Separated rats fed *B. breve* DPC6330 (C), Maternally Separated rats fed *B. breve* DPC6330 (D), Non-Maternally Separated rats fed *B. breve* DPC6330 plus 0.5% (w/w) linoleic acid and 0.5% (w/w) α-linolenic acid (E), Maternally Separated rats fed *B. breve* DPC6330 plus 0.5% (w/w) linoleic acid and 0.5% (w/w) α-linolenic acid (F) for 7 weeks.

g/100 g FAME	Adipose Tissue					
	A	B	C	D	E	F
**C16:0**	24.26±0.34	23.33±0.47	24.32±0.54	24.42±0.35	23.36±0.27	22.76±0.49
**C16:1c9**	3.51±0.15	^c*^ 2.79±0.20	^b*^ 3.74±0.24	3.44±0.16	3.20±0.20	3.08±0.23
**C18:0**	4.15±0.11	4.40±0.08	4.07±0.09	4.32±0.17	4.45±0.12	3.99±0.04
**C18:1c9**	^b,f***^ 25.80±0.13	^a,c**^ 24.77±0.21	^b**,f***^ 25.97±0.27	^f**^ 25.52±0.22	25.13±0.20	^a***,c**,d**^ 24.24±0.24
**C18:1** ***t*** **9**	1.78±0.06	1.66±0.04	1.56±0.08	1.69±0.06	1.75±0.04	1.58±0.06
**C18:2** ***n*** **-6**	35.02±0.60	37.40±0.78	34.68±0.89	35.04±0.58	35.99±0.69	37.64±0.81
**C18:3** ***n*** **-3**	^e***,f***^ 2.10±0.05	^f***^, 2.27±0.06	^e**, f***^ 2.14±0.06	^e**, f***^ 2.15±0.06	^a***,c***,d***,f***^ 2.66±0.13	^a***,b***,c***,d***,e**^ 3.31±0.17
**C20:1** ***c*** **11**	^b*^ 0.208±0.007	^a*,d*^ 0.232±0.004	0.214±0.005	^b*^ 0.207±0.005	0.213±0.005	0.216±0.004
**C20:4** ***n*** **-6**	0.48±0.04	0.48±0.03	0.54±0.05	0.51±0.04	0.42±0.01	0.46±0.02

Results are expressed as percentage of total identified fatty acids. Data are Means ± SEM g/100 g FAME.

^A, B, C, D, E, F^ Different superscript letters within a column indicate significant difference (* = p<0.05, ** = p<0.01, *** = p<0.001). FAME = fatty acid methyl esters. C16:0 palmitic acid; C16:1c9 palmitoleic acid; C18:0 stearic acid; C18:1*c*9 oleic acid; C18:1*t*9 elaidic acid**;** C18:2n-6 linoleic acid; C18:3n-3 linolenic acid; C20:1*c*11 eicosenoic acid; C20:4n-6 arachidonic acid.

### The effect of *B. breve* DPC6330±0.5% (w/w) linoleic acid and 0.5% (w/w) α-linolenic acid on the fatty acid composition of rat serum

After 7 weeks of dietary treatment, the DHA content of the MS control group was lowest of all six groups, with a significant increase in DHA content of the NS control group (p<0.05, Table 3). The NS rats receiving *B. breve* (without LA and ALA supplementation) exhibited an increased serum concentration of EPA compared to un-supplemented rats (p<0.05, Table 3). The same effect was not observed in MS rats, however. Maternally separated animals receiving *B. breve* DPC6330 in combination with fatty acid supplementation exhibited significantly increased serum EPA concentration compared to the *B. breve* DPC6330 supplemented MS group (p<0.001, Table 3). The mean DPA content of the serum of NS rats was significantly increased following *B. breve* DPC6330 supplementation and *B. breve* DPC6330 supplementation in combination with LA and ALA (p<0.01 and p<0.05, respectively, Table 3). Oral administration of *B. breve* to MS rats did not result in a change the DPA content of the serum, however, administration of *B. breve* DPC6330 in combination with fatty acid supplementation did significantly increase DPA, compared to the MS control group (p<0.01, Table 3). The palmitoleic acid content of the serum of the MS control group was highest of all six groups, with a significant decrease in palmitoleic acid content of the NS control group compared to the MS control group (p<0.05, Table 3).

**Table 3:** Fatty acid profile in serum of Non-Maternally Separated rats fed un-supplemented diet (A), Maternally Separated rats fed un-supplemented diet (B), Non-Maternally Separated rats fed *B. breve* DPC6330 (C), Maternally Separated rats fed *B. breve* DPC6330 (D), Non-Maternally Separated rats fed *B. breve* DPC6330 plus 0.5% (w/w) linoleic acid and 0.5% (w/w) α-linolenic acid (E), Maternally Separated rats fed *B. breve* DPC6330 plus 0.5% (w/w) linoleic acid and 0.5% (w/w) α-linolenic acid (F) for 7 weeks.

g/100 g FAME	Serum					
	A	B	C	D	E	F
**C16:0**	22.61±0.19	23.43±0.26	23.46±0.25	22.97±0.25	22.94±0.20	23.38±0.20
**C16:1c9**	^b*^ 1.15±0.08	^a*^ 1.85±0.11	1.45±0.12	1.29±0.12	1.53±0.13	1.39±0.11
**C18:0**	11.68±0.23	11.29±0.28	11.86±0.20	11.45±0.24	11.26±0.26	11.87±0.16
**C18:1c9**	11.07±0.22	11.42±0.39	10.68±0.22	11.70±0.25	11.32±0.33	10.98±0.18
**C18:1** ***t*** **9**	1.58±0.03	1.79±0.04	1.70±0.05	1.58±0.05	1.69±0.07	1.63±0.04
**C18:2** ***n*** **-6**	28.65±0.31	27.98±0.53	27.93±0.50	28.34±0.48	28.82±0.44	28.08±0.47
**C18:3** ***n*** **-3**	1.26±0.04	1.39±0.03	^e*^ 1.18±0.04	1.26±0.07	^c*^ 1.45±0.06	1.29±0.07
**C20:3n-6**	0.41±0.01	0.44±0.02	0.44±0.01	0.45±0.01	0.41±0.01	0.45±0.02
**C20:4** ***n*** **-6**	15.38±0.36	14.29±0.55	15.22±0.27	14.54±0.46	14.81±0.49	14.42±0.28
**C20:5** ***n*** **-3**	^c*,f**^ 0.19±0.01	0.21±0.01	^a*,d*^ 0.23±0.007	^c,f***^ 0.18±0.006	^f*^ 0.20±0.008	^a**,d***,e^ 0.25±0.02
**C22:4** ***n*** **-6**	0.29±0.02	0.32±0.01	0.33±0.01	0.33±0.01	0.28±0.02	0.30±0.02
**C22:5** ***n*** **-3**	^c**,e*,f***^ 0.341±0.01	^f**^ 0.359±0.02	^a**, d*^ 0.414±0.01	^c*,f***^ 0.355±0.01	^a*^ 0.412±0.01	^a***,b**, d***^ 0.443±0.02
**C22:6** ***n-3***	^b*^ 1.83±0.09	^a*^ 1.42±0.08	1.66±0.12	1.74±0.12	1.60±0.11	1.71±0.07

Results are expressed as percentage of total identified fatty acids. Data are Means ± SEM g/100 g FAME.

^A, B, C, D, E, F^ Different superscript letters within a column indicate significant difference (* = p<0.05, ** = p<0.01, *** = p<0.001). FAME = fatty acid methyl esters. C16:0 palmitic acid; C16:1c9 palmitoleic acid; C18:0 stearic acid; C18:1*c*9 oleic acid; C18:1*t*9 elaidic acid; C18:2n-6 linoleic acid; C18:3n-3 linolenic acid; C20:3n-6 dihomo-γ-linolenic acid; C20:4n-6 arachidonic acid; C20:5n-3 eicosapentaenoic acid; C22:4 adrenic acid; C22:5n-3 docosapentaenoic acid; C22:6n-3 docosahexaenoic acid.

### The effect of *B. breve* DPC6330±0.5% (w/w) linoleic acid and 0.5% (w/w) α-linolenic acid on the fatty acid composition of rat prefrontal cortex

The palmitoleic acid content of the prefrontal cortex of the MS control group was highest of all six groups (0.63 ± 0.04 g/100 g FAME), with a significant decrease in palmitoleic acid content of the *B. breve* DPC6330 administered MS group (p<0.05, 0.41 ± 0.01 g/100 g FAME, Table 4).

**Table 4:** Fatty acid profile in prefrontal cortex of Non-Maternally Separated rats fed un-supplemented diet (A), Maternally Separated rats fed un-supplemented diet (B), Non-Maternally Separated rats fed *B. breve* DPC6330 (C), Maternally Separated rats fed *B. breve* DPC6330 (D), Non-Maternally Separated rats fed *B. breve* DPC6330 plus 0.5% (w/w) linoleic acid and 0.5% (w/w) α-linolenic acid (E), Maternally Separated rats fed *B. breve* DPC6330 plus 0.5% (w/w) linoleic acid and 0.5% (w/w) α-linolenic acid (F) for 7 weeks.

g/100 g FAME	Prefrontal Cortex					
	A	B	C	D	E	F
**C16:0**	26.89±0.45	25.24±0.46	25.54±0.52	26.22±0.33	25.11±0.50	25.14±0.42
**C16:1c9**	0.53±0.03	^d*^ 0.63±0.04	0.53±0.03	^b*^ 0.41±0.01	0.54±0.05	0.55±0.04
**C18:0**	23.72±0.20	23.44±0.20	23.50±0.30	23.42±0.14	23.37±0.10	23.28±0.07
**C18:1c9**	14.16±0.14	13.95±0.11	14.01±0.21	14.04±0.17	14.01±0.17	14.15±0.20
**C18:1** ***t*** **9**	2.79±0.06	2.97±0.06	2.96±0.05	2.86±0.04	2.91±0.05	2.95±0.05
**C18:2** ***n*** **-6**	0.89±0.08	0.79±0.07	0.87±0.04	0.87±0.05	0.85±0.04	0.97±0.05
**C20:4** ***n*** **-6**	10.75±0.22	11.28±0.25	11.34±0.17	11.08±0.11	11.46±0.18	11.54±0.11
**C22:4**	3.47±0.08	3.63±0.08	3.56±0.08	3.56±0.04	3.57±0.07	3.57±0.04
**C24:1**	1.24±0.04	1.37±0.06	1.29±0.05	1.22±0.04	1.21±0.05	1.28±0.04
**C22:6** ***n-3***	13.57±0.31	13.75±0.26	13.72±0.25	14.23±0.29	14.37±0.27	14.20±0.16

Results are expressed as percentage of total identified fatty acids. Data are Means ± SEM g/100 g FAME.

^A, B, C, D, E, F^ Different superscript letters within a column indicate significant difference (* = p<0.05, ** = p<0.01, *** = p<0.001). FAME = fatty acid methyl esters. C16:0 palmitic acid; C16:1c9 palmitoleic acid; C18:0 stearic acid; C18:1*c*9 oleic acid; C18:1*t*9 elaidic acid; C18:2n-6 linoleic acid; C20:4n-6 arachidonic acid; C22:4 adrenic acid; C24:1 nervonic acid; C22:5n-3 docosapentaenoic acid; C22:6n-3 docosahexaenoic acid.

## Discussion

This study shows that there are differences in arachidonic acid, DHA, ALA and palmitoleic acid in the tissues of NS and MS rats and indeed the levels of palmitoleic acid, arachidonic acid and DHA can be influenced by oral administration of *B. breve* DPC6330 in MS rats. The present study supports previous observations that altering the gut microbiota changes host fat composition [47], [48], [49]. Indeed, the strain has previously been found to alter the palmitoleic acid content in the brain and adipose tissue, DHA in the liver and adipose tissue, increased propionate in the caecum and alterations the caecal microbiota in C57BL6/J mice [49]. It is clear that the strain is metabolically active in the GI tract, however, no increases in arachidonic acid and DHA were observed in the liver of NS rats fed *B. breve* DPC6330. Interestingly, we have previously seen that feeding *B. breve* DPC6330 to MS and NS rats elicited differing responses, however, in that case *B. breve* DPC6330 altered brain derived neurotrophic factor (BDNF) in the NS animals, with no effect in MS rats [52]. We suggested that the reason a response was not observed in MS rats following administration of *B. breve* DPC6330 was due to a ceiling effect for the biomarker and perhaps this is the same in relation to palmitoleic acid, arachidonic acid and DHA for NS rats fed *B. breve* DPC6330. *B. breve* DPC6330 had a greater effect on fatty acid metabolism in MS animals compared with NS animals. There were only two instances in this study where feeding *B. breve* DPC6330 to the NS rats significantly increased fatty acid content (EPA and DPA in the serum, both are involved in the pathway for DHA production and have a number of proposed health benefits [53]). Recently EPA, rather than DHA, has been identified as the key ω-3 fatty acid in treating depression [54]. EPA was only detected in measurable amounts in the serum of animals throughout the study. Administering *B. breve* DPC6330 to NS rats increased EPA but did not influence EPA in MS rats. Feeding *B. breve* DPC6330 plus fatty acid supplementation to NS rats did not change EPA levels compared to un-supplemented controls, it did however, increase EPA levels in the serum of MS animals compared to the MS *B. breve* DPC6330 fed group. This is another example of the different responses in NS and MS rats. Feeding *B. breve* DPC6330 to MS rats also significantly decreased the amount of eicosenoic acid (*c*11) in the adipose tissue, reaching similar levels as observed in the NS rats.

The mechanism by which *B. breve* DPC6330 changes fatty acid composition observed in the present study is uncertain and remains to be elucidated. Solakivi *et al*. suggested that decreased arachidonic acid and DHA in humans with IBS may by due to reduced intestinal mal-absorption rather than reduced biosynthesis by desaturases and elongases [41]. However, in the case of *B. breve* DPC6330 feeding, perhaps the strain aids in intestinal absorption of PUFAs or regulates desaturase activity, as has previously been suggested [47], since probiotic bacteria have been reported to influence desaturase activity in animal tissues and increase serum arachidonic acid [55]. Perhaps feeding *B. breve* DPC6330 to rats influences corticosterone levels, a stress related hormone involved in fat metabolism [56]. Previously corticosterone has been reported to be elevated in MS rats [25], [57] and indeed, feeding probiotics reduced stress induced corticosterone levels [57], [58]. Fatty acid supplementation in this study did not influence the arachidonic acid or DHA contents in rat tissues or serum, it did however, increase the ALA acid content in the adipose tissue, as expected. Feeding *B. breve* DPC6330 in combination with fatty acid supplementation significantly increased the EPA content in the serum of MS rats, compared with *B. breve* DPC6330-fed MS rats but not the MS control group, while also increasing the DPA content in MS rats compared with un-supplemented and *B. breve* DPC6330-fed animals. It is likely that fatty acid supplementation had little effect on the fatty acid content of tissues and serum of the rats due to the fact that the animal diet already contained high levels of LA and ALA (3.1% (w/w) and 0.3% (w/w), respectively).

Arachidonic acid and DHA are crucial factors in brain development, neurogenesis and neurotransmission, and also influence cognitive processes in the brain [59], [60], [61] and as a result are incorporated into infant formula [62]. The anti-inflammatory properties of EPA and DHA and their ability to decrease proinflammatory cytokines have previously been reported [63], [64], [65]. It has been suggested that the anti-inflammatory properties of EPA and DHA are predominantly due to the replacement of arachidonic acid in cell membranes, resulting in decreased production of arachidonic acid-derived proinflammatory eicosanoids such as prostaglandin E_2_ and leukotriene B_4_ [63]. However, in this study we found the arachidonic acid content of the liver increased in association with the DHA content in response to *B. breve* DPC6330 feeding in the MS animals. This shows that arachidonic acid is not being replaced, as the content of both arachidonic acid and DHA are increasing. It has previously been reported that female human patients with IBS have elevated levels of plasma arachidonic acid, linked to an increase in eicosanoid production [39]. Furthermore, an increase in plasma arachidonic acid has been observed in MS rats when compared to NS rats, as well as increases and decreases in stearic and oleic acids, respectively [29]. More recently, conflicting results have found the levels of arachidonic acid and DHA to be lower in the serum of IBS patients, varying results were not attributed to gender differences [41]. Similarly, we found increased liver arachidonic acid and serum DHA levels in NS rats compared with MS rats. The differences maybe due to differences in samples assayed (serum v plasma).

It has previously been reported that probiotic administration decreased visceral hypersensitivity [66] and changes in the species and numbers of the faecal microbiota have been reported in IBS patients [67]. Significant differences in the fatty acid content of tissues were only detected for the palmitoleic and elaidic acid of the adipose tissue of the MS control and *B. breve* DPC6330 fed NS groups, the groups with the greatest differences in pain observed thresholds. A significant difference was also recorded in the palmitoleic acid contents in the serum of NS and MS controls and in the liver and prefrontal cortex of MS control and MS rats fed *B. breve* DPC6330. An increase in palmitoleic acid (not significant) has previously been seen in IBS patients compared to controls [41]. Interestingly, palmitoleic acid has recently been described as a “lipokine”, i.e. an adipose tissue-derived lipid hormone which links adipose tissue to systemic metabolism [68]. It has been linked with suppression of adipocyte cytokine expression, promotion of pancreatic β-cell proliferation, enhancement of skeletal muscle glucose uptake and stimulation of adipocyte peroxisome proliferator–activated receptor-c transcriptional activity in animal studies [68], [69], [70], [71], however, its effects on human health are still unclear [72]. Clearly, there are differences in the serum levels of palmitoleic acid in MS and NS rats and perhaps it plays a role in IBS, however, more research is needed to confirm this. This ability of bifidobacteria to influence the fatty acid content in MS rats is another example of a potential therapeutic application of the genus to treat IBS and depression, a list which already includes normalization of the immune response, reversal of behaviour deficits and the restoration of basal noradrenaline concentrations [30].

In conclusion, our results demonstrate that 1) there are differences in arachidonic acid, DHA, ALA and palmitoleic acid in the tissues of NS and MS rats, 2) palmitoleic acid, arachidonic acid and DHA can be influenced by oral administration of *B. breve* DPC6330 in MS rats, 3) palmitoleic acid plays a role in maternally separated rats, however, further studies are required to draw conclusions about the role of this fatty acid in IBS patients.
